# Patient experiences of an online consultation system: a qualitative study in English primary care post-COVID-19

**DOI:** 10.3399/BJGP.2023.0076

**Published:** 2024-03-19

**Authors:** Susan Moschogianis, Sarah Darley, Tessa Coulson, Niels Peek, Sudeh Cheraghi-Sohi, Benjamin C Brown

**Affiliations:** School of Health Sciences, Health Services Research and Primary Care, University of Manchester, Manchester.; School of Health Sciences, Health Services Research and Primary Care, University of Manchester, Manchester.; NHS Salford Clinical Commissioning Group, Salford.; Imaging and Data Science, University of Manchester, Manchester Academic Health Science Centre, Manchester; NIHR Greater Manchester Patient Safety Translational Research Centre, School of Health Sciences, University of Manchester, Manchester.; School of Health Sciences, Health Services Research and Primary Care, University of Manchester, Manchester; NIHR Greater Manchester Patient Safety Translational Research Centre, School of Health Sciences, University of Manchester, Manchester.; School of Health Sciences, Health Services Research and Primary Care, University of Manchester, Manchester; and chief medical officer, Patchs Health, London.

**Keywords:** e-visit, general practice, online consultation, primary health care, qualitative research, remote consultation, teleconsultation

## Abstract

**Background:**

Online consultation systems (OCSs) allow patients to contact their healthcare teams online. Since 2020 they have been rapidly rolled out in primary care following policy initiatives and the COVID-19 pandemic. In-depth research of patients’ experiences using OCSs is lacking.

**Aim:**

Explore patients’ experiences of using an OCS.

**Design and setting:**

Qualitative study in English GP practices using the Patchs OCS (www.Patchs.ai) from March 2020 to July 2022.

**Method:**

Thematic analysis of 25 patient interviews and 21 467 written comments from 11 851 patients who used the OCS from nine and 240 GP practices, respectively.

**Results:**

Patients cited benefits of using the OCS as speed, flexibility, and efficiency. Nevertheless, some patients desired a return to traditional consultation methods. GP practices often did not clearly advertise the OCS or use it as patients expected, which caused frustration. Patients reported advantages of having a written record of consultations and the opportunity to communicate detailed queries in free text. Views differed on how the OCS influenced clinical safety and discussions of sensitive topics. Patients who struggled to communicate in traditional consultations often preferred using the OCS, and male patients reported being more likely to use it.

**Conclusion:**

Globally, this is the largest in-depth study of patient experiences of an OCS. It contributes new knowledge that the patient experience of using OCSs can be influenced by previously unreported patient characteristics and the conditions they consult about. Further, it contributes recommendations on the design and implementation of the OCS in practice.

## Introduction

Online consultation systems (OCSs) are digital communication tools that allow patients to request help from their healthcare team over the internet^1^ by completing free text^2^ or multiple-choice questionnaire forms.^3^ Practitioners can resolve patient queries by written message, telephone call, video consultation, or by arranging an in-person visit.^1^^,^^4^

Internationally, OCSs have been promoted as a way to relieve pressure on healthcare services through more effective use of resources.^5^^–^^8^ Their adoption was catalysed by the COVID-19 pandemic as a way for patients to receive care remotely and reduce the risk of spreading the virus.^9^ English GP practices have been mandated to offer OCSs since April 2020 as part of a ‘Digital first primary care’ policy.^10^ During the COVID-19 pandemic practices were advised to conduct ‘Total digital triage’^11^ where all initial patient contacts were made via an OCS. Since May 2020, OCSs have been available in 85% of English GP practices,^12^ and, in those, using OCSs as their primary method of patient contact is estimated to account for 72% of all incoming patient requests.^13^

Outside England, OCSs are also used in the rest of the UK, and research has been published on their use in Canada, Denmark, the Netherlands, New Zealand, Norway, Scotland, Spain, Sweden, and the US.^14^^–^^29^ This international research shows that OCSs can have positive impacts on care, such as increased access, though can have conflicting impacts on costs, workload, patient satisfaction, and care equity.

Research into experiences using OCSs has been largely conducted pre-COVID-19, and predominantly among clinicians.^17^^,^^18^^,^^30^^–^^32^ Exploration of patient views has been limited^33^^,^^34^ with most studies employing survey-based methods,^24^^,^^28^^,^^35^^–^^38^ rather than qualitative interview-based techniques. Findings have often been hampered by low patient uptake or usage by specific patient groups such as younger patients.^2^^,^^32^^,^^39^ Given the increased adoption of OCSs since the COVID-19 pandemic, there is a need to explore patient experiences of using them in depth.^34^

### Online consultation system

Patchs functions in the same way as all OCSs: it is accessed online from the GP practice website and allows patients to request help from their GP practice in writing. Patients can submit clinical requests, for example, a new health problem or an ongoing health problem; and administrative requests, for example, a fit note or medication. A specific feature of Patchs is that patients describe their requests by providing unstructured free-text responses to open-ended questions in a ‘chatbot’, as opposed to filling in a multiple-choice questionnaire. It is estimated that approximately 13% of OCSs allow patients to solely describe their requests using free text.^34^ Chatbot questions are fixed, though vary according to the type of request selected by patients. Questions cover topics of typical traditional primary care consultations, including a description of the request, and patients’ ideas, concerns, and expectations.^40^ Staff aim to respond to patients within a stipulated timeframe set by the GP practice — for example, 48 working hours, either by written message, video/telephone call, or by arranging an in-person visit. Supplementary Box S1 provides a full description of the system using the Template for intervention description and replication (TIDieR) checklist.^41^

**Table:** How this fits in

Online consultation systems (OCSs) have been rolled out rapidly, but little is known about patients’ experiences when using them. This study was the largest ever reported qualitative study of patient experiences using an OCS. The findings provide insight into why some patients prefer in-person consultations, and why others prefer to use an OCS. Patients’ experiences of using OCSs can be influenced by how they are designed, how GP practices use them, patient characteristics, and the type of request.

This study aimed to explore patients’ experiences using OCSs since the COVID-19 pandemic. Through an existing research collaboration, this study focused on one OCS called Patchs (Patchs Health, London; www.Patchs.ai), which has been available to GP practices in England since December 2019. Specifically, the researchers sought to explore why patients chose to use the OCS, the circumstances in which they preferred to use it, and the perceived facilitators and barriers to its use. The authors anticipate the findings will be relevant to other OCSs therefore adding value for all GP practices using OCSs and other OCS designers.

## Method

The researchers conducted a qualitative study using semi-structured interviews triangulated with large volumes of written feedback to explore patients’ experiences of using the specified OCS named Patchs.

### Setting and sample

A sample of GP practices (*n* = 240) were included in the study from March 2020 to July 2022: London (*n* = 153), Southeast England (*n* = 12), North West England (*n* = 44), South England (*n* = 25), Midlands (*n* = 4), and Wales (*n* = 2). All GP practices that used the OCS during the study period were included.

### Data collection

A total of 25 semi-structured interviews were conducted by telephone by either of the first three authors. All patients submitting feedback, both positive and negative, were asked if they consented to being contacted by a member of the research team to take part in an interview about their experience. Interviewees were recruited via email; sampling was purposive and maximum variation was sought for factors that could influence OCS experience including age, sex, and ethnicity^34^ (Table 1)*.* The interview guide explored the strengths, weaknesses, opportunities, and threats of OCSs in general, such as online access and written communication, using Patchs as an example (Supplementary Box S2). Interviews were audio-recorded and transcribed verbatim.

**Table 1.** Participant characteristics

**Characteristic**	**Patients interviewed (*n* = 25)**	**Patient feedback contributors (*n* = 11 851)**
**Sex**		
Male : female : not specified, *n* (%)	13 (52) : 12 (48) : 0 (0)	4176 (35.3) : 7642 (64.5) : 33 (<1)

**Age, years**		
Mean (SD)	51.7 (±16.12)	46.21 (±19.51)
Range	20 to 75	18 to 99

**Ethnicity, *n* (%)**		
Asian or Asian British — Indian	2 (8)	485 (4)
Asian or Asian British — Pakistani	1 (4)	182 (2)
Asian or Asian British — Bangladeshi	0	174 (1)
Asian (other)	0	363 (3)
Black or Black British — Caribbean	0	399 (3)
Black or Black British — African	0	629 (5)
Black or Black British (other)	0	93 (1)
Chinese	0	70 (1)
White British	20 (80)	3839 (32)
White Irish	—	166 (1)
White (other)	2 (8)	1061 (9)
Mixed	—	369 (3)
Other ethnic groups	—	397 (3)
Not known	—	3624 (31)

**Occupation, *n* (%)**		
Employed	16 (64)	Unknown
Unemployed	2 (8)	
Retired	5 (20)	
Student	1 (4)	
Unknown	1 (4)	

**Frequency of OCS use**	**Number of times used OCS**	**Number of times feedback left**

Median, *n* (IQR)	5 (7–2)	1 (49–1)
Range (minimum to maximum)	1 to 22	1 to 106

**Other characteristics, *n* (%)^a^**		
Speak English as a second language	5 (20)	Unknown
Carer	3 (12)	
Older person (≥65 years)	7 (28)	
Hearing impaired	1 (4)	
Long-term condition(s)	5 (20)	
Asylum seeker	2 (8)	

a *Groups are not mutually exclusive so do not add up to 100%. IQR = interquartile range. OCS = online consultation system. SD = standard deviation.*

Written free-text feedback was obtained from all 240 practices and included 21 467 comments from 11 851 patients. Feedback was collected in the OCS using the question: ‘How was your experience using Patchs today?’ in a free-text box of unlimited character count (‘Please tell us more in the box below’). Patients were prompted to provide feedback after each interaction with the OCS — for example when submitting a request, replying to a message, or on completion of a consultation. Patients could also provide feedback at any time by accessing a ‘Give feedback’ link in the OCS. Supplementary Box S3 provides further information on data collection.

### Data analysis

Interview transcripts and written feedback comments were imported into NVivo (version 12). Using thematic analysis,^42^ codes were inductively developed and transcripts independently coded line by line by at least two out of three primary authors. Findings were triangulated between interviews and written comments using a convergence coding matrix that displayed data from both sources enabling data to be compared and contrasted.^43^ Data analysis was conducted alongside data collection; both ceased at saturation when themes were fully developed with clear definitions, and no new information emerged after at least three interviews.^42^ Supplementary Box S3 provides further information on data analysis.

## Results

The GP practices (*n* = 240) had used the OCS for a range of 1 to 31 months by the end of the study.

In total 230 patients were invited to participate and interviews were conducted on all those who responded (*n* = 25; 11%). The semi-structured interviews that were conducted with 25 patients had an average duration of 38.5 minutes (range 16 to 62 minutes) and were from nine GP practices who had used the OCS for 14.4 months on average (range 6 to 20 months; Supplementary Table S1) at time of interviews.

Findings from interviews and written feedback were similar and are reported together with example quotes from both data sources. Additional quotes are shown in Supplementary Table S2.

### Perceived benefits of the online consultation system

Most patients identified advantages of the OCS over traditional methods of contacting their GP practice, especially for ‘simple’ health problems such as ‘rashes’ or ‘colds’. The primary benefit reported by most patients was the ability to receive a quick response to their query, compared with the delay (typically 2–3 weeks) associated with traditional routine in-person appointments. The convenience and flexibility of the OCS in terms of how and when it could be accessed, particularly for patients who worked full time or had childcare considerations, was also advantageous. Patients believed the OCS saved them time by avoiding busy phone lines to reception, unnecessary travel, and long waits due to delayed appointments:

*‘The advantages to me were it was fairly quick, there was less wait than there would be if I had to go to the surgery, or had to make an appointment. It was more instantaneous.’*

(Interview, practice [PR] 2, patient [P] 2, male [M ], aged 74 years)

The ability to provide their GP with detailed information in advance, should a telephone or in-person consultation be necessary, was also felt to be efficient:

*‘I mean, it saves me time, as well, because like everybody, I’m time poor, so being able to do this online and then have a chat with the doctor when they phone me back, and I’ve already given them all the details they need — I think it works very well for me. I think it’s brilliant.’*

(Interview, Pr1, P3, M, aged 61 years)

Despite the perceived benefits of the OCS, patients often had negative experiences. However, these were largely related to how the GP practice implemented and used the OCS, rather than the design of the OCS itself. This is explored in detail in the next section.

### GP practice implementation of the online consultation system

#### Communication about the service

Poor communication about the OCS often left patients disappointed and frustrated. Some practices adopted a ‘soft launch’ where the OCS was not available to all patients initially owing to fear of being inundated. When made more widely available, patients were often not notified in advance, and only learned about the OCS from reception staff at the point they were unwell and needed help. GP practice websites were frequently criticised for being overloaded with information, and needed clearer signposting to the OCS.

Some patients were confused about the purpose of the OCS, particularly when their practice offered other online patient-facing systems with different functions, for example, to order prescriptions. Some patients were unaware that in-person appointments would be offered if necessary, whereas others thought the OCS was an in-person appointment booking service:

*‘I’m not sure what the purpose of it is … I’m not sure if it’s a by-pass in terms of appointments … That was never really clear to me … did I need to still ring up?’*

(Interview, Pr5, P13, female [F], aged 40 years)

#### Expectation versus reality

Patients expected certain events to happen after submitting a request. If it did not happen it negatively impacted their experience, which led to mistrust and reluctance to use the OCS again. Patients expected to receive acknowledgement from the practice of their OCS request soon after submission, and for it to be fully resolved within the timeframe they advertised, for example, 48 working hours. If this did not happen, they often telephoned the GP practice to chase a response. Patients expressed irritation when the GP had not read the details of their request before an in-person or telephone consultation, which dissuaded them from providing in-depth information in future:

*‘The problem on both occasions hasn’t been the logging of information from my side. It’s what happens with that information when it goes to the GP practice … Neither of them* [GPs] *accessed the notes I’d put on* [the OCS]*. They had no idea what the consultation was going to be about … It’s put me off putting any detail in.’*

(Interview, Pr7, P22, F, aged 45 years)

Patients expected their request to be actioned appropriately and informed of how their request was resolved. They also wanted the opportunity to ask follow-up questions if anything was unclear:

‘*In this instance the case has just been closed off by the reception team, however the feedback I have does not fully help me so now I can either raise another request explaining the whole matter again or call the surgery which is what I thought the site was trying to prevent.’*

(Feedback, Pr5, F, aged 38 years)

A small number of feedback patients preferred that a particular doctor resolve their request. However, all interviewees reported they did not see the same GP before their practice’s implementation of the OCS anyway. Most patients reported that they valued speed of response over continuity of care:

‘*Perhaps a question could be, do you want the request dealt with by the same doctor or by the one who can deal with it the quickest?’*

(Feedback, Pr53, M, aged 75 years)

#### Access management

The way practices managed patient demand through the OCS varied significantly, and was a prominent theme in the feedback data. Many GP practices limited the availability and number of requests that could be submitted by patients each day to avoid being overwhelmed. Uptake of the OCS was high and demand almost always exceeded these limits. This meant patients could often not access the OCS when they wanted to, thus removing its benefit of flexibility:

*‘It excludes people who have jobs and responsibilities as not everyone has access to the internet at seven thirty in the morning.’*

(Feedback, Pr18, F, aged 23 years)

However, some GP practices limited availability of the OCS with minimal impact on patient experience. These practices were clear about when the OCS was available, had staggered opening times throughout the day, had higher limits on request numbers, and allowed administrative request submissions when closed for clinical queries.

#### ‘Personal’ service

Many patients reported the OCS had a positive impact on the relationship with their GP practice. Key to positive experiences was the perception of a ‘personal’ service, which included staff acknowledging patient concerns, swift replies to requests, and direct contact from a GP — either by written message or telephone:

*‘Even the doctor herself got involved in the delivery of the advice and the guidance. Either by picking up the phone directly or by … messaging through the system* [it] *worked very well.’*

(Interview, Pr2, P6, M, aged 58 years)

Though the OCS was embraced at the start of the pandemic, feedback comments showed some patients believed they were a temporary response to COVID-19. These patients were frustrated and expected default in-person consultations to return:

*‘I am trying to reach out to my GP but these online services are making it impossible … these GPs are now hiding behind their computers and patients are being ignored and made to feel helpless.’*

(Feedback, Pr61, F, aged 62 years)

These patients found the OCS impersonal and felt detached from their GP practice. This was particularly evident in practices using ‘Total digital triage’,^11^ where patients often felt forced into using the OCS. They were disappointed they could no longer chat to reception staff freely and felt that in-person appointments would become increasingly rare:

*‘I’m concerned there could be a tendency for it to be so successful from their side that face-to-face appointments be less of an option.’*

(Interview, Pr6, P18, F, aged 57 years)

#### Support and guidance

Though most patients said the OCS was easy to use, those lacking confidence with technology in general felt empowered when they received support by practice staff.

### Written communication between patient and GP practice

#### Written record

Almost all patients felt it was beneficial to have a written record of communication with their GP practice. They often found it difficult to remember information given verbally in traditional consultations, and liked the ability to refer back later:

*‘I prefer it, because it’s written down again. Verbally you might not take it all in at the time. So, you’ve got that physical hard copy to keep.’*

(Interview, Pr2, P9, M, aged 39 years)

#### Opportunity to communicate detailed queries in free text

Though some patients found the chatbot questions impersonal, most thought they were useful prompts that mirrored conversations with clinicians in traditional consultations. Patients liked the ability to respond to the questions using unstructured free text and describe concerns in their own words. Some patients had used other OCSs that used multiple-choice questionnaires, which they found to be restrictive, complicated, and time-consuming:

*‘This* [OCS] *so far looks very good as it is easier and more efficient than* [previous multiple-choice questionnaire system]*.* [Previous system] *asked many unnecessary questions that had no relevance to the consultation, but this is direct and easy to use asking specific questions.’*

(Feedback, Pr21, M, aged 34 years)

Patients liked the ability to write, review, and edit queries in their own time. They compared the OCS to traditional in-person appointments, which were time limited. Patients often felt rushed during traditional consultations and worried they would forget to share key information. Others felt the OCS questions compelled them to provide more detailed information:

*‘You can go over what you’ve written and edit as you need to, or think, oh no, I need to elaborate on that point a bit, whereas if you were face-to-face, then I don’t know. You would probably just, in my case, just stick your foot out and say, have a look.’*

(Interview, Pr1, P3, M, aged 61 years)

#### Clinical safety

Some patients were concerned that clinicians may overlook important information delivered verbally during in-person or telephone consultations. These patients felt the OCS was safer:

*‘I prefer* [OCS] *because everything is recorded. While sometimes when you’re face-to-face there are things that you say that it’s not being recorded as said … You always check, oh, did I remember to say that?’*

(Interview, Pr1, P1, F, aged 48 years)

A minority of patients feared that using the OCS may misdirect GPs to an incorrect diagnosis. They believed the OCS placed responsibility on them to share key information in sufficient detail. Others worried the lack of non-verbal cues and wider physical context of in-person appointments could result in serious issues being overlooked:

*‘That would be the danger I would feel, that the doctor makes an assessment presumably how the doctor sees you. Does she see you as well in general or does she spot something else that’s maybe wrong. You go in with your big toe and she may think, mmm, something doesn’t look quite right there in some other area.’*

(Interview, Pr5, P16, M, aged 73 years)

### Preference for online consultation system use according to patient and request characteristics

#### Sensitive topics

Some patients felt in-person consultations were most appropriate for ‘sensitive’ topics such as mental and sexual health issues. They believed the pre-set chatbot questions were impersonal and uncaring, and that patients may be forced to articulate emotional topics twice — initially via the OCS, and then again verbally in a telephone or in-person consultation:

*‘I think part of the benefit of face-to-face is that you have the conversations and quite often they bring out other aspects. So with* [the OCS] *I think you might find it quite difficult to deal with issues like mental health because it’s quite hard to explain in those boxes and within those questions …’*

(Interview, Pr5, P13, F, aged 40 years)

In contrast, some patients felt anxious when discussing sensitive topics on the telephone and in-person, and preferred to use the OCS precisely *because* it was impersonal. When using the OCS they felt detached and less embarrassed, meaning they could communicate more openly:

*‘You know, you might be embarrassed or something like that, with mental health issues and stuff like that, you might not necessarily work good seeing someone face-to-face, and you can actually get your point across a bit better writing it down.’*

(Interview, Pr7, P21, M, aged 35 years)

#### Patients with communication challenges

Patients who struggled with verbal communication in traditional telephone or in-person appointments usually found it easier to use the OCS. This included patients with hearing loss, anxiety, and autistic spectrum disorders. Patients for whom English was a second language often preferred the OCS because they were more confident writing rather than speaking English, and had access to free online tools to support them:

*‘If I was face to face, I would probably have to do a lot of paraphrasing … having the doctor guessing sometimes what I mean. Whereas now that it’s typing I can just use, I don’t know, the* [online automatic translation service] *or the* [health website] *for similar symptoms and see what, not particularly the medical term but maybe the more or less slang term, if that makes sense.’*

(Interview, Pr2, P7, F, aged 38 years)

In contrast, feedback indicated that some patients with attention deficit hyperactivity disorder (ADHD) found the OCS difficult to use owing to the written nature of the communication:

*‘I have ADHD … I also do not have the time or patience and often even skill to sign into* [OCS] *to retrieve messages … I don’t want to have to type my flipping issues when I am distressed and needing to speak with a doctor.’*

(Feedback, Pr68, F with ADHD, aged 41 years)

#### Male patients

Male patients reported they were more likely to contact their GP practice using the OCS. Some were concerned they were wasting GP time and believed OCSs were more efficient for practices. Others admitted to delaying contacting their GP practice as an avoidance tactic to cope with anxiety about an underlying serious diagnosis. The impersonal nature of the OCS, and the act of writing down their concerns, helped them emotionally detach:

*‘I’m always reluctant to go to the doctor, I almost hope things will clear up if I leave it a week or so … I’m slightly distanced here, I’m a bit worried about this and maybe there’s something wrong … I can do that a little removed and it cushions me from having actually to ring the surgery or go to the surgery and say, oh, I’m worried.’*

(Interview, Pr5, P16, M, aged 73 years)

#### Older patients

A common preconception was that OCSs would exclude older patients. Though initially apprehensive about the OCS, many older participants found the system easier to navigate than expected and often preferred using it to contact their GP practice than traditional methods.

## Discussion

### Summary

This qualitative in-depth study explored the experience of patients using an OCS in English primary care during and after the peak of the COVID-19 pandemic when uptake was high, and is the first and largest of its kind.

Overall, patients had positive experiences using the OCS, though this was influenced by how GP practices used them, the design of the technology, characteristics of the patient, and clinical condition they used the OCS for. In general, patients believed the OCS was quicker, more flexible, and more efficient than traditional consulting methods. Despite this, some still wanted a return to traditional ways of accessing their GP practice via telephone and in-person visits. There was substantial variation in how GP practices implemented and used the OCS, which was often the key driver of patient experience. Most patients welcomed the ability to communicate with their GP practice in free-text writing using the OCS, though views differed on whether this had positive or negative impacts on clinical safety. Some patients preferred discussing sensitive topics using the OCS, whereas others preferred in-person consultations. Patients who struggled to communicate verbally in traditional GP consultations found it easier to use the OCS, whereas those with attention disorders found it more difficult. Male patients reported being more likely to contact their GP practice using the OCS, and many older patients found the system easy to use. Further research is needed to identify which patients did not use the OCS and why.

This study provides new insights into why some patients may prefer to use OCSs or traditional ways of contacting their GP practice. GP practices should focus on clear communication about OCSs, good practice OCS ‘etiquette’, and manage OCS availability fairly.

### Strengths and limitations

This study generated rich narratives from patients by triangulating qualitative data from in-depth interviews and large volumes of written feedback. Globally, qualitative research studies exploring patient experiences of using OCSs before the COVID-19 pandemic were often limited by low levels of adoption or usage by specific patient groups, such as younger patients.^2^^,^^32^^,^^37^^,^^39^^,^^44^ This study was conducted after the start of the COVID-19 pandemic when GP practices implemented the OCS into their daily workflows and saw high patient uptake. Patients in the present study had typically used the OCS multiple times and came with a broad range of characteristics, including ethnic minority backgrounds, patients who speak English as a second language, and older people, thus providing insights into factors affecting high adoption that have not been previously reported,^2^^,^^32^^,^^39^ such as access management and evolving attitudes towards OCSs over time. Though only a small proportion of participants who were approached agreed to be interviewed (11%), participants had varied experiences of the OCS. Patients who left feedback comments had both positive and negative experiences (Supplementary Box S3), therefore inviting them to be interviewed after leaving feedback could be viewed as a strength of the recruitment method.

A potential limitation of this study was that patient experience was evaluated using only one OCS. To counteract this the researchers focused on reporting findings related to functions shared by all OCSs, such as online access, and written communication between patients and their healthcare teams, rather than features unique to Patchs. Additionally, some participants had used other OCSs and were able to compare and contrast their experiences of using different features, such as describing their requests as free text versus multiple-choice questionnaires. A further potential limitation was that the experiences of patients who did not use the OCS was not explored. This may have provided further insights into barriers to adopting the OCS, and its broader impacts on patient care.

### Comparisons with existing literature

Consistent with pre-COVID-19 research conducted internationally, the authors found overall high patient satisfaction with OCSs^2^^,^^3^^,^^15^^,^^21^^,^^28^^,^^36^^,^^37^^,^^39^^,^^45^^–^^48^ and varying views on their safety.^14^^,^^18^^,^^21^^,^^27^^,^^28^^,^^44^^,^^47^^,^^49^ However, they also found a subgroup of patients with an evolving negative attitude towards their ongoing use after the pandemic peak. These patients wanted a return to traditional in-person appointments by default, citing concerns about clinical safety and a lack of personal connection to their GP practice. Some, however, could not articulate specific reasons why in-person consultations were preferable. These views may have been fuelled by rhetoric in the British media and the government that GP practices were not conducting in-person appointments during the COVID-19 pandemic.^50^^,^^51^

Prior qualitative research has identified advantages for specific groups of patients when using OCSs, including those with hearing loss,^2^ anxiety,^38^ work commitments, or care responsibilities,^35^ all of which are corroborated by the present findings. This study found new evidence that male patients and those with autistic spectrum disorder may also benefit from using OCSs, though the latter will likely only benefit if the condition does not also impact written communication skills.^52^ This study further identified that patients with ADHD are potentially a new group of patients that may find using OCSs difficult.

Previous qualitative research has also shown that GP practice staff have concerns that older patients^2^ and those who speak English as a second language^35^ may find OCSs difficult to use. This appears to be supported by quantitative research that shows these patient groups use OCSs less frequently than younger patients and native English language speakers.^48^ The results from the study presented in this article provide new insights that these groups can still derive benefits from OCSs, and that some may actually prefer using them to traditional consultation methods.

### Implications for research and practice

This study found that how GP practices used the OCS and aspects of its design were key drivers of patient experience. Drawing directly from the present findings, the authors make specific recommendations for GP practices and OCS designers on how they can optimise patients’ experience when using OCSs (Box 1 and Box 2, respectively).

**Box 1.** Recommendations for GP practices using online consultation systems to optimise patient experience

**Clearly communicate to patients about the service**
Let them know *when* an online consultation system (OCS) is launched, and *how* it can be accessed. OCSs are typically accessed via the GP practice website, so they should be prominent and easy to find. Be clear what the OCS should be used for, including whether in-person appointments will be arranged if necessary — particularly if other online services are also available.
**Practise good OCS ‘etiquette’**
Send a brief acknowledgement message to patients to confirm the practice has received their request as soon as possible, and then contact patients within the stated timeframe. Read the request first — do not ask patients to repeat what they have already written. Action the request and inform the patient how their request was resolved. Offer the opportunity for patients to ask questions. This will encourage patients to provide detail in future requests, and avoid them telephoning the practice to chase responses and submitting duplicate queries.
**Manage OCS access fairly**
If availability and number of OCS requests patients can submit is limited, communicate clearly to patients when OCSs will be available, and stagger opening times throughout the day, for example, not just at 8:00 am. Maximise the number of queries the practice will accept, and allow administrative queries to be submitted when closed for clinical ones — for example, requests for fit notes and repeat prescriptions.
**Support patients who struggle to use OCSs**
By providing support on the telephone if needed.
**Provide a ‘personal’ service**
Strengthen the relationship with patients by acknowledging their concerns, replying quickly to their queries, facilitating direct contact with a GP where appropriate, for example, by phone or written message and arranging in-person appointments if preferred.
**Make other options available**
Some patients will not want to use an OCS to discuss specific conditions — for example, sensitive topics — and some may struggle to use them because of pre-existing diagnoses — for example, attention deficit hyperactivity disorder. Some may not have a clear reason for not wanting to use OCSs. Other options to access the GP practice, such as telephone or in-person appointments, should be available.
**Promote OCSs to patients who may traditionally struggle to access primary care**
Some patient groups who traditionally struggle to access primary care may derive specific advantages from using OCSs, such as those with hearing loss, anxiety, and autistic spectrum disorder.
**Do not make assumptions about who may not want to use an OCS**
Certain patients may counterintuitively prefer to use OCSs, such as older people and those who speak English as a second language. Enable patients to use OCSs when they would prefer.

**Box 2.** Recommendations for online consultation system designers to optimise patient experience

**Allow patients to write their queries using free text**
Patients who had previously used multiple-choice questionnaires reported them to be restrictive and time consuming. This study found that patients liked the ability to openly describe their problems in detail using their own words.
**Allow patients to ask follow-up questions**
A common complaint was the perception that the patient’s request was closed prematurely. This resulted in a reluctance to trust the OCS in the future. In this study, patients frequently requested the ability to clarify responses from their GP practice and to ask further questions before their request was closed.
**Manage expectations**
Patients often reported confusion as to the purpose of how the GP practice was using the OCS and were then disappointed if it did not meet their expectations. OCS providers should explain clearly to patients what OCSs should (and should not) be used for, how their queries may be resolved by their GP practice — for example, by arranging in-person appointments — and how to get help if OCSs are temporarily unavailable.

NHS guidance recommends that free online translation tools should not be used in clinical practice because their accuracy cannot be verified.^53^ However, the present results show that patients will often use them without the knowledge of their GP practice; further consideration for how this could be managed by GP practices and OCS designers is required.

Because of the increasing complexity and available features in OCSs,^34^ future research should compare different OCS features and GP practice implementations to ascertain which approaches are most effective, as well as investigating novel designs that may enhance patient experience. To understand the wider impacts of OCSs on patient care, research is needed to evaluate the experiences of patients who do not use OCSs at practices where they are available.

## References

[1] Bakhai M, Atherton H (2021). How to conduct written online consultations with patients in primary care. BMJ.

[2] Eccles A, Hopper M, Turk A, Atherton H (2019). Patient use of an online triage platform: a mixed-methods retrospective exploration in UK primary care. Br J Gen Pract.

[3] Player M, O’Bryan E, Sederstrom E (2018). Electronic visits for common acute conditions: evaluation of a recently established program. Health Aff.

[4] (2019). Primary Care Strategy and NHS Contracts Group. Digital-first primary care.

[5] NHS (2019). The NHS long term plan.

[6] Gill M (2011). A national telehealth strategy for Australia — for discussion.

[7] Daniel H, Sulmasy LS, Health and Public Policy Committee of the American College of Physicians (2015). Policy recommendations to guide the use of telemedicine in primary care settings: an American College of Physicians position paper. Ann Intern Med.

[8] Hobbs FD, Bankhead C, Mukhtar T (2016). Clinical workload in UK primary care: a retrospective analysis of 100 million consultations in England, 2007–14. Lancet.

[9] Neves AL, Li E, Gupta PP (2021). Virtual primary care in high-income countries during the COVID-19 pandemic: policy responses and lessons for the future. Eur J Gen Pract.

[10] NHS England GP Contract documentation 2019/20. https://www.england.nhs.uk/gp/investment/gp-contract/gp-contract-documentation-2019-20/2019.

[11] NHS England (2020). Advice on how to establish a remote ‘total triage’ model in general practice using online consultations. https://www.england.nhs.uk/coronavirus/documents/advice-on-how-to-establish-a-remote-total-triage-model-in-general-practice-using-online-consultations/.

[12] Bakhai M (2020). The use of online and video consultations during the COVID-19 pandemic — delivering the best care to patients. https://transform.england.nhs.uk/blogs/use-online-and-video-consultations-during-covid-19-pandemic-delivering-best-care-patients/.

[13] Clarke GM, Dias A, Wolters A (2022). Access to and delivery of general practice services: a study of patients at practices using digital and online tools.

[14] Landgren S, Cajander Å (2021). Non-use of digital health consultations among Swedish elderly living in the countryside. Front Public Health.

[15] Nilsson E, Sverker A, Bendtsen P, Eldh AC (2021). A human, organization, and technology perspective on patients’ experiences of a chat-based and automated medical history-taking service in primary health care: interview study among primary care patients. J Med Internet Res.

[16] Penza KS, Murray MA, Myers JF (2020). Management of acute sinusitis via e-visit. Telemed J E Health.

[17] Eldh AC, Sverker A, Bendtsen P, Nilsson E (2020). Health care professionals’ experience of a digital tool for patient exchange, anamnesis, and triage in primary care: qualitative study. JMIR Hum Factors.

[18] Entezarjou A, Bolmsjö BB, Calling S (2020). Experiences of digital communication with automated patient interviews and asynchronous chat in Swedish primary care: a qualitative study. BMJ Open.

[19] Fernández OS, Seguí FL, Vidal-Alaball J (2020). Primary care doctor characteristics that determine the use of teleconsultations in the Catalan public health system: retrospective descriptive cross-sectional study. JMIR Med Inform.

[20] Johansson A, Larsson M, Ivarsson B (2020). General practitioners’ experiences of digital written patient dialogues: a pilot study using a mixed method. J Prim Care Community Health.

[21] Kelley LT, Phung M, Stamenova V (2020). Exploring how virtual primary care visits affect patient burden of treatment. Int J Med Inform.

[22] Seguí FL, Aguilar RAG, de Maeztu G (2020). Teleconsultations between patients and healthcare professionals in primary care in Catalonia: the evaluation of text classification algorithms using supervised machine learning. Int J Environ Res Public Health.

[23] Stamenova V, Agarwal P, Kelley L (2020). Uptake and patient and provider communication modality preferences of virtual visits in primary care: a retrospective cohort study in Canada. BMJ Open.

[24] Zanaboni P, Fagerlund AJ (2020). Patients’ use and experiences with e-consultation and other digital health services with their general practitioner in Norway: results from an online survey. BMJ Open.

[25] Fagerlund AJ, Holm IM, Zanaboni P (2019). General practitioners’ perceptions towards the use of digital health services for citizens in primary care: a qualitative interview study. BMJ Open.

[26] Bertelsen P, Stub Petersen L (2015). Danish citizens and general practitioners’ use of ICT for their mutual communication. Stud Health Technol Inform.

[27] Bishop TF, Press MJ, Mendelsohn JL, Casalino LP (2013). Electronic communication improves access, but barriers to its widespread adoption remain. Health Aff.

[28] Cowie J, Calveley E, Bowers G, Bowers J (2018). Evaluation of a digital consultation and self-care advice tool in primary care: a multi-methods study. Int J Environ Res Public Health.

[29] Wilson G, Currie O, Bidwell S (2021). Empty waiting rooms: the New Zealand general practice experience with telehealth during the COVID-19 pandemic. N Z Med J.

[30] Fagerlund AJ, Holm IM, Zanaboni P (2019). General practitioners’ perceptions towards the use of digital health services for citizens in primary care: a qualitative interview study. BMJ Open.

[31] Banks J, Farr M, Edwards H (2018). Use of an electronic consultation system in primary care: a qualitative interview study. Br J Gen Pract.

[32] Casey M, Shaw S, Swinglehurst D (2017). Experiences with online consultation systems in primary care: case study of one early adopter site. Br J Gen Pract.

[33] Mold F, Hendy J, Lai Y-L, de Lusignan S (2019). Electronic consultation in primary care between providers and patients: systematic review. JMIR Med Inform.

[34] Darley S, Coulson T, Peek N (2022). Understanding how the design and implementation of online consultations affect primary care quality: systematic review of evidence with recommendations for designers, providers, and researchers. J Med Internet Research.

[35] Farr M, Banks J, Edwards HB (2018). Implementing online consultations in primary care: a mixed-method evaluation extending normalisation process theory through service co-production. BMJ Open.

[36] Johansson A, Larsson M, Ivarsson B (2020). Patients’ experiences with a digital primary health care concept using written dialogues: a pilot study. J Prim Care Community Health.

[37] Salisbury C, Ipsos MORI, York Health Economics Consortium (2019). Evaluation of Babylon GP at hand: final evaluation report. NHS Hammersmith and Fulham CCG and NHS England.

[38] Carter M, Fletcher E, Sansom A (2018). Feasibility, acceptability and effectiveness of an online alternative to face-to-face consultation in general practice: a mixed-methods study of webGP in six Devon practices. BMJ Open.

[39] Turner A, Morris R, Rakhra D (2022). Unintended consequences of online consultations: a qualitative study in UK primary care. Br J Gen Pract.

[40] Larsen J-H, Neighbour R (2014). Five cards: a simple guide to beginning the consultation. Br J Gen Pract.

[41] Hoffmann TC, Glasziou PP, Boutron I (2014). Better reporting of interventions: template for intervention description and replication (TIDieR) checklist and guide. BMJ.

[42] Braun V, Clarke V (2006). Using thematic analysis in psychology. Qual Res Psychol.

[43] Farmer T, Robinson K, Elliott SJ, Eyles J (2006). Developing and implementing a triangulation protocol for qualitative health research. Qual Health Res.

[44] Atherton H, Brant H, Ziebland S (2018). Alternatives to the face-to-face consultation in general practice: focused ethnographic case study. Br J Gen Pract.

[45] Zanaboni P, Fagerlund AJ (2020). Patients’ use and experiences with e-consultation and other digital health services with their general practitioner in Norway: results from an online survey. BMJ Open.

[46] Peber E, Wästfelt E (2020). Impact of digi-physical healthcare.

[47] McGrail KM, Ahuja MA, Leaver CA (2017). Virtual visits and patient-centered care: results of a patient survey and observational study. J Med Internet Res.

[48] Atherton H, Brant H, Ziebland S (2018). The potential of alternatives to face-to-face consultation in general practice, and the impact on different patient groups: a mixed-methods case study.

[49] Johansson A, Larsson M, Ivarsson B (2020). Patients’ experiences with a digital primary health care concept using written dialogues: a pilot study. J Prim Care Community Health.

[50] Oliver D (2021). *Daily Mail*’s campaign on general practice won’t help GPs or their patients. BMJ.

[51] Mroz G, Papoutsi C, Rushforth A, Greenhalgh T (2021). Changing media depictions of remote consulting in COVID-19: analysis of UK newspapers. Br J Gen Pract.

[52] Hofvander B, Delorme R, Chaste P (2009). Psychiatric and psychosocial problems in adults with normal-intelligence autism spectrum disorders. BMC Psychiatry.

[53] NHS England (2018). Guidance for commissioners: interpreting and translation services in primary care.

